# Progress in Bacteriology

**Published:** 1901-01-12

**Authors:** 


### PROGRESS IN BACTERIOLOGY.

**Immunity.**—Ehrlich's theory of immunity[1] is as follows:—The proteid molecule consists of a central closely-welded nucleus, to which are attached other complex radicles or "side chains." Poisons may unite with these side-chains, alter them, and so alter the original molecule. Poisons (toxins) uniting with side chains upset the equilibrium of the molecule in the cell of which it forms a part and new side-chains are formed to replace the old; the new side-chains become free in the circulation, meet more toxins and unite with them. In fact they are antitoxin. A given poison can only unite with side-chains of a given and corresponding structure; that is to say the action of all serums is specific. In the case of any given disease in an individual, natural immunity implies absence of suitable side-chains; high susceptibility the possession of many suitable side-chains. To secure active immunity we have so to stimulate the cells so as to lead to the formation of many side-chains; for passive immunity it is only necessary to introduce side-chains from without (antitoxins), the immunity passing off as the antitoxin is gradually eliminated. The agglutinating reaction of certain sera is due to the presence of alexins, bodies which are capable of promoting the solution of any kind of cell and of bacteria. Antistreptococcus serum should be fresh and obtained from an animal immunised against several varieties of streptococci. Antipneumococcus serum has no effect beyond possibly preventing a general blood infection.

**The Vitality of Germs.**—Macfadyen and Rowland[2] have experimented with a view to determining the effects of extreme cold on bacterial life. The temperature of liquid air (–190°C) had no appreciable effect upon the vitality of micro-organisms, nor had that of liquefied hydrogen (–252°C). The organisms experimented upon were B. typhosus, diphtheriæ, B. Coli and staphylococcus. The vitality of bacteria buried from 18 to 24 inches below the surface of the ground is however rapidly destroyed, so that the fear that graveyards and cemeteries may become a source of risk to health is unfounded. These points have recently been elucidated by Klein.[3]

**Glycerine as an Antiseptic.**—Pure glycerine has feeble bactericidal powers, but when mixed with 30 to 50 per cent. of water its action is intensified. Most antiseptics are more powerful in watery solution than when mixed with glycerine; and glycerine, when added to antiseptic soaps, tends to render them inert. A 5 per cent. solution of carbolic acid in glycerine is inert until water is added in equal quantity, and this holds good for all solutions of less than 10 per cent. In connection with the above results a series of experiments made by Rosenau[4] on the viability of the plague bacillus are of practical importance. He found that this bacillus could not stand complete desiccation at a temperature of 30°C, but under favourable conditions retained its vitality for seventy-five days, although generally perishing in fourteen days if the temperature approaches 27°C, or if desiccation were carried to an extreme point rapidly. This last point is also demonstrated by Delepine,[5] who finds that, whilst very susceptible to light and heat, the plague bacillus is extremely resistant to cold, retaining its vitality even if kept below freezing point for four months. Pure cultures are killed by direct sunlight in one hour, by a temperature of 80°C. in five minutes, by corrosive sublimate (1 in 1,000) solution instantaneously, and by 5 per cent. carbolic acid solution in one minute. In tap water, urine and sputum their vitality is preserved for from four to five days.

**Tubercle and Smegma Bacilli.**—Pakes[6] has shown that the smegma bacillus is more widely distributed than was formerly thought to be the case, and is frequently mistaken for the tubercle bacillus. Two methods of staining, Honsell's, and a modified Ziehl-Neelsen's, are used to distinguish the organisms.

1. *Modified Ziehl-Neelsen.*—Make films, dry in the air, fix by passing through the flame. Stain in hot carbol-fuchsin for five minutes. Wash in water. Wash in absolute alcohol until completely decolourised, and again wash in water. Immerse in 25 per cent. sulphuric acid for five seconds, wash, and counterstain with methylene blue.

2. *Honsell's Method.*—Stain in warm carbol-fuchsin for five minutes, wash. Immerse for ten minutes in a mixture of absolute alcohol 97 per cent., hydrochloric acid 3 per cent. Wash in water. Counterstain with equal parts of saturated alcoholic solution of methylene blue and water. Wash in water, dry, and mount.

By both of these processes the smegma is decolourised by the alcohol, and the tubercle bacillus appears stained red. Another method of differentiating between the two organisms is given by Dorset, who finds that the smegma bacillus is not stained by Sudan iii, whilst the tubercle bacillus takes on a bright red colour.

**Whooping Cough.** — Luzzatto[7] has studied the bacteriology of this disease from washed specimens of the sputum, and cultures on blood-serum and blood-agar. He describes two organisms as being constantly present, the one a lanceolate diplococcus with polar staining, and the other a long slender bacillus resembling the influenza bacillus.

**A Bacillus of Vaccine Lymph.**—Nakanishi[8] has isolated a bacillus from calf and human vaccine lymph, with the following characters:—It is polymorphous, appearing either as a rod-shaped form showing bipolar staining, or as an oval or round refractive organism similar to that described by Guianieri. The bacillary forms undergo involution changes taking on wedge-shaped or clubbed forms closely resembling the diphtheria bacillus. Cultures may be made on agar or on solidified serum. Successful experimental inoculations were made on the cornea of rabbits, and typical vesicles were produced in two out of four persons whose arms were inoculated.

[1] Brit. Med. Jour., Oct. 13, 1900. [2] Ibid, Oct. 27, 1900. [3] Lancet, May 12, 1900. [4] Pub. Health Rep., U.S. Marine Hosp. Serv. [5] Brit. Med. Jour., Oct. 27, 1900. [6] Ibid, Jan. 27, 1900. [7] Cent. f. Bakt., June 30, 1900. [8] Cent. f. Bakt., Bd. 27, No. 18.

